# (4a*S*,4b*R*,7*R*,10a*S*)-3,7-Dimethyl-10a-(propan-2-yl)-1,4,4a,4b,5,6,7,8,10,10a-deca­hydro­phenanthrene-1,4-dione

**DOI:** 10.1107/S160053681104517X

**Published:** 2011-11-05

**Authors:** Ignez Caracelli, Julio Zukerman-Schpector, André T. Lousada Machado, Timothy J. Brocksom, M. Lúcia Ferreira, Edward R. T. Tiekink

**Affiliations:** a BioMat-Departamento de Física, Universidade Federal de São Carlos, CP 676, 13565-905 São Carlos, SP, Brazil; b Laboratório de Cristalografia, Estereodinâmica e Modelagem Molecular, Universidade Federal de São Carlos, Departamento de Química, CP 676, 13565-905 São Carlos, SP, Brazil; c Universidade Federal de São Carlos, Departamento de Química, CP 676, 13565-905 São Carlos, SP, Brazil; d Department of Chemistry, University of Malaya, 50603 Kuala Lumpur, Malaysia

## Abstract

In the title compound, C_19_H_26_O_2_, the *A* ring adopts a chair conformation, whereas the *B* and *C* rings both adopt distorted half-chair conformations with the quaternary C atom common to both rings lying 0.577 (3) and 0.648 (3) Å out of the approximate plane defined by the remaining five C atoms (r.m.s. deviations = 0.1386 and 0.1156 Å) for the *B* and *C* rings, respectively. Mol­ecules are assembled in the crystal through C—H⋯O inter­actions involving both carbonyl O atoms, which lead to supra­molecular chains aligned along the *b* axis.

## Related literature

For background to the biological activity of some diterpene compounds, see: Guo *et al.* (2011 ▶); Slusarczyk *et al.* (2011 ▶). For the synthesis, see: Ferreira (2002 ▶). For conformational analysis, see: Cremer & Pople (1975 ▶).
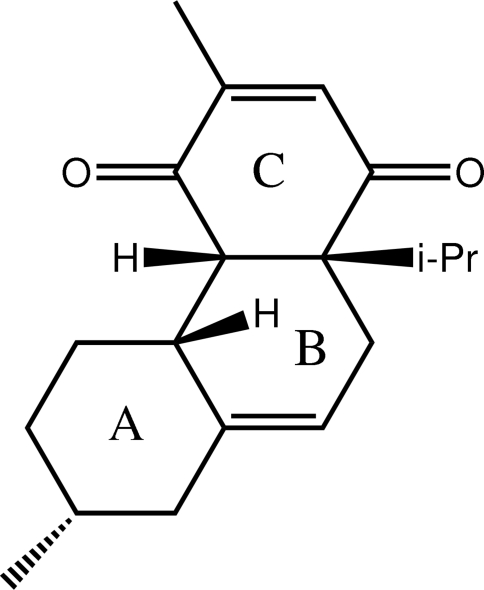


## Experimental

### 

#### Crystal data

C_19_H_26_O_2_
*M* *_r_* = 286.40
Monoclinic, 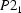

*a* = 10.882 (1) Å
*b* = 6.6015 (9) Å
*c* = 11.656 (1) Å
β = 102.53 (2)°
*V* = 817.40 (16) Å^3^
*Z* = 2
Mo *K*α radiation
μ = 0.07 mm^−1^
*T* = 290 K
0.15 × 0.10 × 0.08 mm

#### Data collection

Enraf–Nonius CAD-4 Mach 3 diffractometer
2470 measured reflections
2334 independent reflections
1100 reflections with *I* > 2σ(*I*)
*R*_int_ = 0.045
3 standard reflections every 30 min  intensity decay: 1.4%

#### Refinement

*R*[*F* ^2^ > 2σ(*F* ^2^)] = 0.037
*wR*(*F* ^2^) = 0.122
*S* = 0.98
2334 reflections
191 parameters
1 restraint
H-atom parameters constrained
Δρ_max_ = 0.18 e Å^−3^
Δρ_min_ = −0.18 e Å^−3^

### 

Data collection: *CAD-4 Software* (Enraf–Nonius, 1989 ▶); cell refinement: *CAD-4 Software*; data reduction: *MolEN* (Fair, 1990 ▶); program(s) used to solve structure: *SIR92* (Altomare *et al.*, 1999 ▶); program(s) used to refine structure: *SHELXL97* (Sheldrick, 2008 ▶); molecular graphics: *ORTEP-3* (Farrugia, 1997 ▶), *DIAMOND* (Brandenburg, 2006 ▶) and *MarvinSketch* (Chemaxon, 2009 ▶); software used to prepare material for publication: *publCIF* (Westrip, 2010 ▶).

## Supplementary Material

Crystal structure: contains datablock(s) global, I. DOI: 10.1107/S160053681104517X/hg5119sup1.cif

Structure factors: contains datablock(s) I. DOI: 10.1107/S160053681104517X/hg5119Isup2.hkl

Supplementary material file. DOI: 10.1107/S160053681104517X/hg5119Isup3.cml

Additional supplementary materials:  crystallographic information; 3D view; checkCIF report

## Figures and Tables

**Table 1:** Hydrogen-bond geometry (Å, °)

*D*—H⋯*A*	*D*—H	H⋯*A*	*D*⋯*A*	*D*—H⋯*A*
C2—H2⋯O2^i^	0.98	2.50	3.443 (3)	161
C5—H5⋯O1^ii^	0.93	2.52	3.438 (4)	171

Symmetry codes: (i) 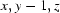; (ii) 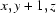.

**Fig. 1.**
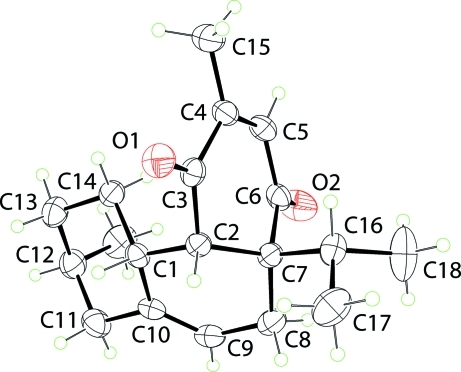
The molecular structure of compound (I) showing atom labelling scheme and displacement ellipsoids at the 30% probability level (arbitrary spheres for the H atoms).

**Fig. 2.**
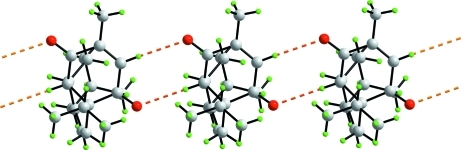
A view of the supramolecular chain in (I) aligned along the b axis and sustained by C—H···O interactions shown as orange dashed lines.

**Fig. 3.**
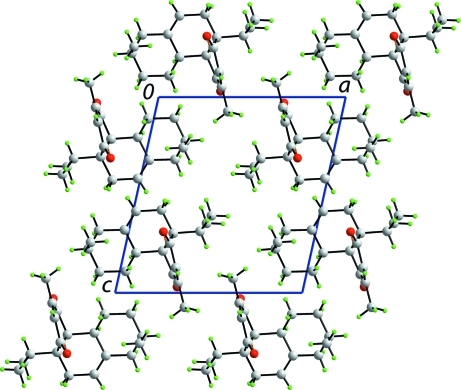
A view in projection down the b axis of the unit-cell contents of (I).

## supplementary crystallographic information

### Comment

Natural diterpenes exhibit a wide range of biological activities such as neuroprotectives (Guo *et al.* 2011) and as antiplasmodials and antitrypanocidals (Slusarczyk *et al.* 2011). While aiming at the synthesis of some hydrophenanthrene diterpenes, a series of new intermediates were obtained and among them, was the title compound (Ferreira, 2002), (I), which has been characterized crystallographically.

In (I), Fig. 1, a chair conformation is found for ring A. Each of the B and C rings presents a distorted half-chair conformation, with the C7 atom, common to both rings, lying 0.5769 (26) and 0.6480 (28) Å, respectively, out of the approximate plane defined by the remaining five C atoms (r.m.s. deviation = 0.1386 and 0.1156 Å for B and C, respectively). The ring puckering parameters for the three rings are: q_2_ = 0.022 (3), 0.315 (3), 0.384 (3) Å, q_3_ = 0.563 (3), -0.273 (3), -0.282 (3) Å, QT = 0.563 (3), 0.417 (3), 0.477 (3) Å, and θ = 2.3 (3), 130.9 (4), 126.3 (4)°, for rings A, B and C, respectively (Cremer & Pople, 1975).

In the crystal packing, the molecules are linked through C–H···O interactions, Table 1, involving both carbonyl-O atoms. This results in the formation of a supramolecular chain along the *b* axis, Fig. 2. The chains pack in the crystal structure with no specific interactions between them, Fig. 3.

### Experimental

The detailed synthesis of the title compound is described in a Ph.D. thesis (Ferreira, 2002). Crystals were grown by slow evaporation from its methanol solution held at 293 K; *M*.pt: 429.6–432.2 K. ^1^H-NMR (CDCl_3_, 400 MHz): δ (p.p.m.) 6.52 (q, 1H, J = 1.5 Hz); 5.35 (d, 1H, J = 3.9 Hz); 3.36 (d, 1H, J = 7.7 Hz); 2.65 (m, 1H); 2.35–2.38 (m, 2H); 2.18–2.19 (m, 2H); 2.01–2.06 (m, 1H); 1.96 (d, 3H, J = 1.5 Hz); 1.90–1.20 (m, 1H); 1.42–1.51 (m, 2H); 1.17–1.27 (m, 2H); 0.94 (d, 3H, J = 6.8 Hz); 0.75 (d, 3H, J = 5.8 Hz); 0.69 (d, 3H, J = 6.8 Hz); ^13^C (CDCl_3_, 100 MHz) δ (p.p.m.) 203.2; 202.6; 149.4; 136.5; 134.3; 118.7; 54.5; 54.2; 42.4; 41.2; 35.9; 32.9; 29.5; 28.0; 21.5; 17.6; 17.4; 16.9; 15.5. Analysis found: C 79.42, H 9.17%. C_19_H_26_O_2_ requires: C 79.68, H 9.15%.

### Refinement

The H atoms were geometrically placed (C–H = 0.93–0.98 Å) and refined as riding with *U_iso_*(H) = 1.2*U_eq_*(C) and *U_iso_*(H) = 1.5*U_eq_*(methyl-C). The absolute structure was based on that of a starting material used in the synthesis (Ferreira, 2002).

### Crystal data

**Table:**

C_19_H_26_O_2_	*F*(000) = 312
*M**_r_* = 286.40	*D*_x_ = 1.164 Mg m^−^^3^
Monoclinic, *P*2_1_	Mo *K*α radiation, λ = 0.71073 Å
Hall symbol: P 2yb	Cell parameters from 25 reflections
*a* = 10.882 (1) Å	θ = 10.3–18.3°
*b* = 6.6015 (9) Å	µ = 0.07 mm^−^^1^
*c* = 11.656 (1) Å	*T* = 290 K
β = 102.53 (2)°	Irregular, colourless
*V* = 817.40 (16) Å^3^	0.15 × 0.10 × 0.08 mm
*Z* = 2	

### Data collection

**Table:**

Enraf–Nonius CAD-4 Mach 3 diffractometer	*R*_int_ = 0.045
Radiation source: fine-focus sealed tube	θ_max_ = 29.0°, θ_min_ = 1.8°
graphite	*h* = −14→14
ω/–2θ scans	*k* = 0→8
2470 measured reflections	*l* = 0→15
2334 independent reflections	3 standard reflections every 30 min
1100 reflections with *I* > 2σ(*I*)	intensity decay: 1.4%

### Refinement

**Table:**

Refinement on *F*^2^	Primary atom site location: structure-invariant direct methods
Least-squares matrix: full	Secondary atom site location: difference Fourier map
*R*[*F*^2^ > 2σ(*F*^2^)] = 0.037	Hydrogen site location: inferred from neighbouring sites
*wR*(*F*^2^) = 0.122	H-atom parameters constrained
*S* = 0.98	*w* = 1/[σ^2^(*F*_o_^2^) + (0.058*P*)^2^] where *P* = (*F*_o_^2^ + 2*F*_c_^2^)/3
2334 reflections	(Δ/σ)_max_ < 0.001
191 parameters	Δρ_max_ = 0.18 e Å^−^^3^
1 restraint	Δρ_min_ = −0.18 e Å^−^^3^

### Special details

**Table:**

Geometry. All s.u.'s (except the s.u. in the dihedral angle between two l.s. planes) are estimated using the full covariance matrix. The cell s.u.'s are taken into account individually in the estimation of s.u.'s in distances, angles and torsion angles; correlations between s.u.'s in cell parameters are only used when they are defined by crystal symmetry. An approximate (isotropic) treatment of cell s.u.'s is used for estimating s.u.'s involving l.s. planes.
Refinement. Refinement of *F*^2^ against ALL reflections. The weighted *R*-factor *wR* and goodness of fit *S* are based on *F*^2^, conventional *R*-factors *R* are based on *F*, with *F* set to zero for negative *F*^2^. The threshold expression of *F*^2^ > 2σ(*F*^2^) is used only for calculating *R*-factors(gt) *etc*. and is not relevant to the choice of reflections for refinement. *R*-factors based on *F*^2^ are statistically about twice as large as those based on *F*, and *R*- factors based on ALL data will be even larger.

### Fractional atomic coordinates and isotropic or equivalent isotropic displacement parameters (Å^2^)

**Table:**

	*x*	*y*	*z*	*U*_iso_*/*U*_eq_	
C1	0.0639 (2)	0.5986 (4)	0.7953 (2)	0.0384 (6)	
H1	0.0437	0.4610	0.8167	0.046*	
C2	0.2045 (2)	0.5917 (4)	0.7858 (2)	0.0343 (6)	
H2	0.2203	0.4553	0.7591	0.041*	
C3	0.2885 (2)	0.6188 (4)	0.9059 (2)	0.0370 (6)	
C4	0.3242 (2)	0.8281 (5)	0.9474 (3)	0.0410 (7)	
C5	0.2941 (3)	0.9818 (5)	0.8724 (2)	0.0432 (7)	
H5	0.3120	1.1128	0.9005	0.052*	
C6	0.2337 (3)	0.9551 (4)	0.7467 (2)	0.0415 (7)	
C7	0.2397 (3)	0.7433 (4)	0.6959 (2)	0.0372 (7)	
C8	0.1491 (3)	0.7223 (5)	0.5767 (2)	0.0471 (7)	
H8A	0.1565	0.8416	0.5300	0.057*	
H8B	0.1741	0.6062	0.5361	0.057*	
C9	0.0148 (3)	0.6975 (5)	0.5837 (2)	0.0467 (7)	
H9	−0.0461	0.7233	0.5159	0.056*	
C10	−0.0248 (2)	0.6416 (4)	0.6784 (2)	0.0422 (7)	
C11	−0.1614 (3)	0.6269 (6)	0.6815 (3)	0.0566 (8)	
H11A	−0.1813	0.4878	0.6972	0.068*	
H11B	−0.2119	0.6641	0.6052	0.068*	
C12	−0.1952 (3)	0.7642 (6)	0.7752 (3)	0.0559 (9)	
H12	−0.2815	0.7323	0.7813	0.067*	
C13	−0.1084 (3)	0.7170 (6)	0.8930 (3)	0.0537 (8)	
H13A	−0.1243	0.5799	0.9159	0.064*	
H13B	−0.1274	0.8083	0.9520	0.064*	
C14	0.0305 (2)	0.7376 (5)	0.8899 (2)	0.0437 (7)	
H14A	0.0819	0.7022	0.9661	0.052*	
H14B	0.0485	0.8771	0.8733	0.052*	
C15	0.3875 (3)	0.8537 (6)	1.0739 (2)	0.0571 (9)	
H15A	0.4098	0.9934	1.0890	0.086*	
H15B	0.4621	0.7718	1.0914	0.086*	
H15C	0.3311	0.8124	1.1225	0.086*	
C16	0.3807 (3)	0.7097 (5)	0.6859 (2)	0.0479 (7)	
H16	0.4338	0.7277	0.7647	0.057*	
C17	0.4060 (3)	0.4981 (6)	0.6445 (3)	0.0711 (11)	
H17A	0.3798	0.3992	0.6947	0.107*	
H17B	0.4943	0.4827	0.6475	0.107*	
H17C	0.3597	0.4788	0.5653	0.107*	
C18	0.4232 (4)	0.8654 (8)	0.6055 (4)	0.0969 (16)	
H18A	0.4079	0.9995	0.6311	0.145*	
H18B	0.3769	0.8457	0.5263	0.145*	
H18C	0.5115	0.8488	0.6086	0.145*	
C19	−0.1910 (4)	0.9882 (7)	0.7419 (3)	0.0748 (11)	
H19A	−0.2468	1.0110	0.6671	0.112*	
H19B	−0.1068	1.0240	0.7372	0.112*	
H19C	−0.2169	1.0700	0.8005	0.112*	
O1	0.32371 (19)	0.4746 (3)	0.96994 (17)	0.0523 (6)	
O2	0.1854 (2)	1.0979 (3)	0.6885 (2)	0.0685 (7)	

### Atomic displacement parameters (Å^2^)

**Table:**

	*U*^11^	*U*^22^	*U*^33^	*U*^12^	*U*^13^	*U*^23^
C1	0.0447 (15)	0.0296 (14)	0.0405 (14)	−0.0061 (13)	0.0087 (11)	0.0004 (13)
C2	0.0455 (15)	0.0231 (14)	0.0352 (13)	0.0008 (13)	0.0106 (12)	−0.0005 (12)
C3	0.0402 (15)	0.0341 (16)	0.0387 (14)	0.0034 (13)	0.0130 (12)	0.0045 (14)
C4	0.0362 (13)	0.0428 (18)	0.0443 (16)	−0.0067 (13)	0.0093 (12)	−0.0088 (15)
C5	0.0481 (16)	0.0291 (15)	0.0515 (16)	−0.0075 (15)	0.0089 (13)	−0.0057 (16)
C6	0.0456 (15)	0.0309 (16)	0.0481 (17)	−0.0071 (14)	0.0100 (13)	0.0028 (15)
C7	0.0477 (15)	0.0298 (16)	0.0355 (14)	−0.0007 (13)	0.0121 (12)	0.0008 (12)
C8	0.0636 (19)	0.0435 (18)	0.0357 (14)	0.0031 (16)	0.0135 (13)	0.0008 (14)
C9	0.0567 (18)	0.0395 (17)	0.0395 (15)	−0.0014 (14)	0.0005 (14)	−0.0069 (14)
C10	0.0456 (16)	0.0366 (17)	0.0421 (15)	−0.0070 (13)	0.0041 (12)	−0.0100 (13)
C11	0.0468 (17)	0.061 (2)	0.0570 (18)	−0.0093 (17)	0.0007 (14)	0.0006 (18)
C12	0.0418 (15)	0.069 (2)	0.0583 (19)	−0.0028 (16)	0.0130 (14)	0.0004 (18)
C13	0.0484 (17)	0.064 (2)	0.0515 (17)	0.0008 (17)	0.0178 (13)	0.0047 (17)
C14	0.0427 (15)	0.0497 (19)	0.0393 (15)	−0.0011 (14)	0.0103 (12)	−0.0004 (14)
C15	0.0494 (17)	0.070 (2)	0.0501 (18)	−0.0090 (18)	0.0060 (14)	−0.0125 (18)
C16	0.0486 (17)	0.0531 (19)	0.0451 (15)	0.0001 (16)	0.0170 (13)	0.0074 (16)
C17	0.076 (2)	0.073 (3)	0.070 (2)	0.021 (2)	0.0293 (18)	−0.007 (2)
C18	0.081 (3)	0.098 (4)	0.127 (4)	0.004 (3)	0.058 (3)	0.046 (3)
C19	0.079 (2)	0.073 (3)	0.075 (2)	0.020 (2)	0.0200 (19)	0.003 (2)
O1	0.0620 (13)	0.0425 (13)	0.0506 (12)	0.0052 (11)	0.0081 (10)	0.0102 (11)
O2	0.0952 (18)	0.0276 (12)	0.0723 (15)	0.0052 (13)	−0.0047 (13)	0.0091 (12)

### Geometric parameters (Å, °)

**Table:**

C1—C10	1.515 (4)	C11—H11B	0.9700
C1—C14	1.538 (4)	C12—C13	1.521 (4)
C1—C2	1.559 (3)	C12—C19	1.532 (6)
C1—H1	0.9800	C12—H12	0.9800
C2—C3	1.507 (4)	C13—C14	1.526 (4)
C2—C7	1.556 (3)	C13—H13A	0.9700
C2—H2	0.9800	C13—H13B	0.9700
C3—O1	1.219 (3)	C14—H14A	0.9700
C3—C4	1.488 (4)	C14—H14B	0.9700
C4—C5	1.333 (4)	C15—H15A	0.9600
C4—C15	1.495 (4)	C15—H15B	0.9600
C5—C6	1.480 (4)	C15—H15C	0.9600
C5—H5	0.9300	C16—C17	1.522 (5)
C6—O2	1.213 (4)	C16—C18	1.528 (5)
C6—C7	1.526 (4)	C16—H16	0.9800
C7—C8	1.525 (4)	C17—H17A	0.9600
C7—C16	1.580 (4)	C17—H17B	0.9600
C8—C9	1.491 (4)	C17—H17C	0.9600
C8—H8A	0.9700	C18—H18A	0.9600
C8—H8B	0.9700	C18—H18B	0.9600
C9—C10	1.322 (4)	C18—H18C	0.9600
C9—H9	0.9300	C19—H19A	0.9600
C10—C11	1.498 (4)	C19—H19B	0.9600
C11—C12	1.524 (5)	C19—H19C	0.9600
C11—H11A	0.9700		
			
C10—C1—C14	109.2 (2)	C13—C12—C11	109.0 (3)
C10—C1—C2	112.7 (2)	C13—C12—C19	112.3 (3)
C14—C1—C2	117.2 (2)	C11—C12—C19	111.6 (3)
C10—C1—H1	105.6	C13—C12—H12	107.9
C14—C1—H1	105.6	C11—C12—H12	107.9
C2—C1—H1	105.6	C19—C12—H12	107.9
C3—C2—C7	111.2 (2)	C12—C13—C14	112.6 (2)
C3—C2—C1	109.7 (2)	C12—C13—H13A	109.1
C7—C2—C1	114.7 (2)	C14—C13—H13A	109.1
C3—C2—H2	106.9	C12—C13—H13B	109.1
C7—C2—H2	106.9	C14—C13—H13B	109.1
C1—C2—H2	106.9	H13A—C13—H13B	107.8
O1—C3—C4	120.0 (2)	C13—C14—C1	110.7 (2)
O1—C3—C2	121.5 (3)	C13—C14—H14A	109.5
C4—C3—C2	118.4 (2)	C1—C14—H14A	109.5
C5—C4—C3	118.9 (2)	C13—C14—H14B	109.5
C5—C4—C15	123.8 (3)	C1—C14—H14B	109.5
C3—C4—C15	117.2 (3)	H14A—C14—H14B	108.1
C4—C5—C6	123.5 (3)	C4—C15—H15A	109.5
C4—C5—H5	118.3	C4—C15—H15B	109.5
C6—C5—H5	118.3	H15A—C15—H15B	109.5
O2—C6—C5	120.4 (3)	C4—C15—H15C	109.5
O2—C6—C7	123.0 (2)	H15A—C15—H15C	109.5
C5—C6—C7	116.5 (2)	H15B—C15—H15C	109.5
C8—C7—C6	111.3 (2)	C17—C16—C18	108.9 (3)
C8—C7—C2	110.5 (2)	C17—C16—C7	113.4 (3)
C6—C7—C2	106.9 (2)	C18—C16—C7	112.3 (3)
C8—C7—C16	111.5 (2)	C17—C16—H16	107.3
C6—C7—C16	106.3 (2)	C18—C16—H16	107.3
C2—C7—C16	110.1 (2)	C7—C16—H16	107.3
C9—C8—C7	114.1 (2)	C16—C17—H17A	109.5
C9—C8—H8A	108.7	C16—C17—H17B	109.5
C7—C8—H8A	108.7	H17A—C17—H17B	109.5
C9—C8—H8B	108.7	C16—C17—H17C	109.5
C7—C8—H8B	108.7	H17A—C17—H17C	109.5
H8A—C8—H8B	107.6	H17B—C17—H17C	109.5
C10—C9—C8	125.3 (3)	C16—C18—H18A	109.5
C10—C9—H9	117.4	C16—C18—H18B	109.5
C8—C9—H9	117.4	H18A—C18—H18B	109.5
C9—C10—C11	122.9 (3)	C16—C18—H18C	109.5
C9—C10—C1	122.9 (2)	H18A—C18—H18C	109.5
C11—C10—C1	114.1 (2)	H18B—C18—H18C	109.5
C10—C11—C12	111.9 (3)	C12—C19—H19A	109.5
C10—C11—H11A	109.2	C12—C19—H19B	109.5
C12—C11—H11A	109.2	H19A—C19—H19B	109.5
C10—C11—H11B	109.2	C12—C19—H19C	109.5
C12—C11—H11B	109.2	H19A—C19—H19C	109.5
H11A—C11—H11B	107.9	H19B—C19—H19C	109.5
			
C10—C1—C2—C3	161.1 (2)	C1—C2—C7—C16	−175.1 (2)
C14—C1—C2—C3	33.1 (3)	C6—C7—C8—C9	−76.9 (3)
C10—C1—C2—C7	35.2 (3)	C2—C7—C8—C9	41.7 (3)
C14—C1—C2—C7	−92.8 (3)	C16—C7—C8—C9	164.6 (2)
C7—C2—C3—O1	−143.0 (3)	C7—C8—C9—C10	−18.3 (4)
C1—C2—C3—O1	89.0 (3)	C8—C9—C10—C11	177.6 (3)
C7—C2—C3—C4	39.3 (3)	C8—C9—C10—C1	1.2 (5)
C1—C2—C3—C4	−88.6 (3)	C14—C1—C10—C9	122.4 (3)
O1—C3—C4—C5	174.1 (3)	C2—C1—C10—C9	−9.7 (4)
C2—C3—C4—C5	−8.2 (4)	C14—C1—C10—C11	−54.3 (3)
O1—C3—C4—C15	−8.5 (4)	C2—C1—C10—C11	173.6 (2)
C2—C3—C4—C15	169.2 (2)	C9—C10—C11—C12	−121.1 (3)
C3—C4—C5—C6	−4.0 (4)	C1—C10—C11—C12	55.6 (4)
C15—C4—C5—C6	178.8 (3)	C10—C11—C12—C13	−54.3 (4)
C4—C5—C6—O2	164.9 (3)	C10—C11—C12—C19	70.3 (4)
C4—C5—C6—C7	−16.9 (4)	C11—C12—C13—C14	56.3 (4)
O2—C6—C7—C8	−15.4 (4)	C19—C12—C13—C14	−67.8 (4)
C5—C6—C7—C8	166.5 (2)	C12—C13—C14—C1	−57.5 (4)
O2—C6—C7—C2	−136.2 (3)	C10—C1—C14—C13	54.0 (3)
C5—C6—C7—C2	45.7 (3)	C2—C1—C14—C13	−176.3 (2)
O2—C6—C7—C16	106.2 (3)	C8—C7—C16—C17	−62.7 (3)
C5—C6—C7—C16	−72.0 (3)	C6—C7—C16—C17	175.8 (2)
C3—C2—C7—C8	−176.7 (2)	C2—C7—C16—C17	60.4 (3)
C1—C2—C7—C8	−51.5 (3)	C8—C7—C16—C18	61.2 (4)
C3—C2—C7—C6	−55.4 (3)	C6—C7—C16—C18	−60.3 (3)
C1—C2—C7—C6	69.8 (3)	C2—C7—C16—C18	−175.7 (3)
C3—C2—C7—C16	59.6 (3)		

### Hydrogen-bond geometry (Å, °)

**Table:**

*D*—H···*A*	*D*—H	H···*A*	*D*···*A*	*D*—H···*A*
C2—H2···O2^i^	0.98	2.50	3.443 (3)	161
C5—H5···O1^ii^	0.93	2.52	3.438 (4)	171

Symmetry codes: (i) *x*, *y*−1, *z*; (ii) *x*, *y*+1, *z*.
